# A Digital Calibration Technique of MEMS Gyroscope for Closed-Loop Mode-Matching Control

**DOI:** 10.3390/mi10080496

**Published:** 2019-07-25

**Authors:** Cheng Li, Bo Yang, Xin Guo, Lei Wu

**Affiliations:** 1 School of Instrument Science and Engineering, Southeast University, Nanjing 210096, China; 2 Key Laboratory of Micro-Inertial Instruments and Advanced Navigation Technology, Ministry of Education, Nanjing 210096, China

**Keywords:** MEMS gyroscope, mode-matching, excitation-calibration, FPGA

## Abstract

A digital excitation-calibration technique of dual-mass MEMS gyroscope for closed-loop mode-matching control is presented in this paper. The technique, which takes advantage of the symmetrical amplitude response of MEMS gyroscope, exploits a two-side excitation signal to actuate the sense mode to obtain the corresponding DC tuning voltage. The structural characteristics of dual-mass decoupled MEMS gyroscope and the tuning principle of excitation-calibration technique are introduced firstly. Then, the scheme of digital excitation-calibration system for the real-time mode-matching control is presented. Simultaneously, open-loop analysis and closed-loop analysis are deduced, respectively, to analyze the sources of tuning error and system stability. To verify the validity of the scheme and theoretical analysis, the system model was established by SIMULINK. The simulation results are proved to be consistent with the theoretical analysis, verifying the feasibility of the digital excitation-calibration technique. The control algorithms of the system were implemented with a FPGA device. Experimental results demonstrate that digital excitation-calibration technique can realize mode-matching within 1 s. The prototype with real-time mode-matching control has a bias instability of 0.813${}^{\circ }$/h and an ARW (Angular Random Walk) of 0.0117${}^{\circ }$/$\sqrt{h}$. Compared to the mode-mismatching condition, the bias instability and ARW are improved by 3.25 and 4.49 times respectively.

## 1. Introduction

The Micro-electromechanical System (MEMS) gyroscope is a kind of miniature inertial sensors which is capable of measuring angular rate. Due to the advantages of small size, low power consumption and high integration, MEMS gyroscopes have been widely adopted for consumer and industrial applications, such as vehicle rollover monitoring, electronic devices rotation detection, industrial robots control, etc. [1,2,3,4,5]. Although initially employed for expensive military applications, current MEMS gyroscopes are still falling behind Fiber Optic Gyroscopes (FOGs), Ring Laser Gyroscopes (RLGs) and other tactical grade gyroscopes in terms of bias stability [3,6]. Thus, how to further improve the performance and explore the potential capabilities of MEMS gyroscope is a hot issue in recent research [7,8,9,10]. Mode-matching technology, which helps enhance the bias stability and mechanical sensitivity of MEMS gyroscope by eliminating the frequency split between the drive and sense resonance modes [11,12,13], has attracted great attention of researchers.

Numerous research groups have initiated the study of mode-matching technology in early stage and several approaches have been proposed, including selectively depositing or etching polysilicon [14], exploiting localized thermal stress to soften the spring constant [15,16] and some other approaches. However, the majority of previous works have some imperfections. Polysilicon precipitation in [14] adjusts the operating modes of micro-gyroscope by mechanically altering the architecture parameters, while applying thermal stress in [15,16] changes the material parameters. Both of the above techniques require manual intervention and ultra-stringent machining accuracy of gyroscope architecture, making them not suitable for real-time adjustment and batch fabrication. Presently, the electrostatic tuning technology is a more effective approach for automatic mode-matching [17,18]. It utilizes electrostatic negative stiffness effect to alter the equivalent stiffness of the sense mode by applying an adjustable DC voltage to the frequency tuning electrodes of MEMS gyroscope [19,20]. Various means have been developed to attain the perfect DC tuning voltage. Evolutionary computation in [21,22] evaluates the frequency split between two resonance modes by fitting Lorentzian curves to experimental data. An obvious disadvantage of this strategy is requiring manual effort. The phase-domain approach in [23,24] uses the drive signal to actuate sense mode and then employs phase-locked loop (PLL) to measure the phase difference between operating modes, thereby recognizing the mode-matching state. However, this method will enhance the difficulty of Coriolis signal detection. Fuzzy and neural network control algorithms are also used to predict tuning voltage in [25,26]. This intelligent control system can fulfill mode-matching within 8 s, but it is a one-time control method which will become invalid when micro-gyroscope undergoes a Coriolis acceleration. Frequency calibration circuit in [27], which is designed as a feedback architecture, utilizes a calibration engine to generate the calibration signal before the front-end and a ΣΔ ADC to apply the tuning voltage. Although the analog complexity has been greatly reduced owing to the use of ΣΔ ADC, this control circuit still requires a considerable consumption of hardware resources. Observation of noise power difference between two bands located around drive-mode resonance frequency for mode-match determination is reported in [28]. This frequency tuning principle resembles to the frequency calibration circuit above, but the ambient noise needs to be strictly controlled to improve the matching accuracy.

This paper provides a digital excitation-calibration technique for closed-loop mode-matching control of MEMS gyroscope. Excitation signal, composed of two channels signals located on both sides of the drive signal, is used to actuate the sense mode of MEMS gyroscope. The DC tuning voltage extracted from the amplitude response can alter the equivalent stiffness of sense mode, thereby achieving mode-matching control. In Section 2, the structural characteristics of dual-mass MEMS gyroscope and frequency tuning principle are introduced. Section 3 presents the design and analysis of mode-matching control system. System simulations are given in Section 4. Then, we present experimental results in Section 5. Conclusions of the entire research are finally proposed in Section 6.

## 2. Dual-Mass MEMS Gyroscope and Frequency Tuning Principle

**Figure 1:**
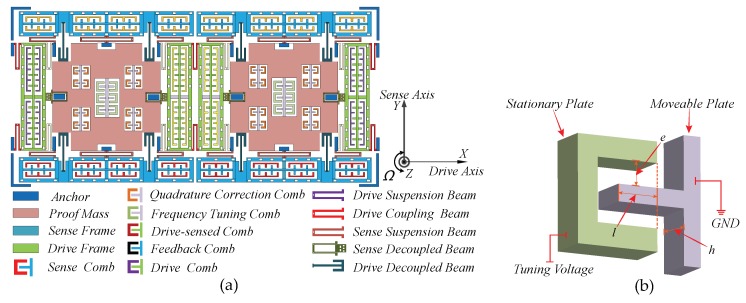
The schematic of the dual-mass MEMS gyroscope: (**a**) the overall structure; and (**b**) the frequency tuning comb.

The dual-mass decoupled MEMS gyroscope consists of two symmetrically arranged substructures [29], as illustrated in Figure 1a. Two substructures are connected by U-shaped drive coupling beams, forcing them to vibrate in the same or reverse direction along the drive axis (*X*-axis). The symmetrical design enables differential detection, which can restrain the common-mode error and improve the Signal to Noise Ratio (SNR) effectively. Owing to the decoupling effect of drive and sense decoupled beams, the quadrature error caused by the cross-talk between harmonic vibrations in drive and sense axes can be effectively suppressed. Nevertheless, because of the fabrication imperfections, the mechanical coupling between drive and sense modes is rather hard to be completely constrained. The residual mechanical coupling will lead to an undesired quadrature error in sense mode. To address the influence of the residual quadrature error on Coriolis signal detection, four groups of correction combs are designed for each substructure. Combined with the dedicated closed control loop, which provides an automatically-adjusted DC voltage to correction combs, the quadrature error can be essentially cancelled. The frequency tuning mechanisms are arranged at the center of each proof mass. As illustrated in Figure 1b, the DC tuning voltage is exerted on the tuning electrode, which connects to the stationary plate. As the potential difference between the moveable and stationary plates raises up, the equivalent stiffness of sense mode will decrease subsequently, thereby tuning sense-mode resonance frequency to approximate that of drive mode and obtaining the maximum sensitivity.

According to the electrostatic negative stiffness effect, when the DC tuning voltage *V${}_{t}$* is applied on the frequency tuning electrodes of MEMS gyroscope, the sense-mode resonance frequency can be simplified as

(1) $$f_{s1}=\frac{\sqrt{{\left(2\pi \times f_{s0}\right)}^{2}-bV_{t}^{2}}}{2\pi }$$

where *f${}_{s0}$* is the natural resonant frequency; *b* is the mechanical parameter and *b* = $\frac{2n\varepsilon hl}{m_{s}e^{3}}$; *n* is the number of tuning combs; *h*, *l* and *e*, respectively, represent the thickness, length and gap of the comb; ε is the vacuum permittivity; and *m${}_{s}$* is the proof mass of sense structure. The design parameters are shown in Table 1.

**Figure 2:**
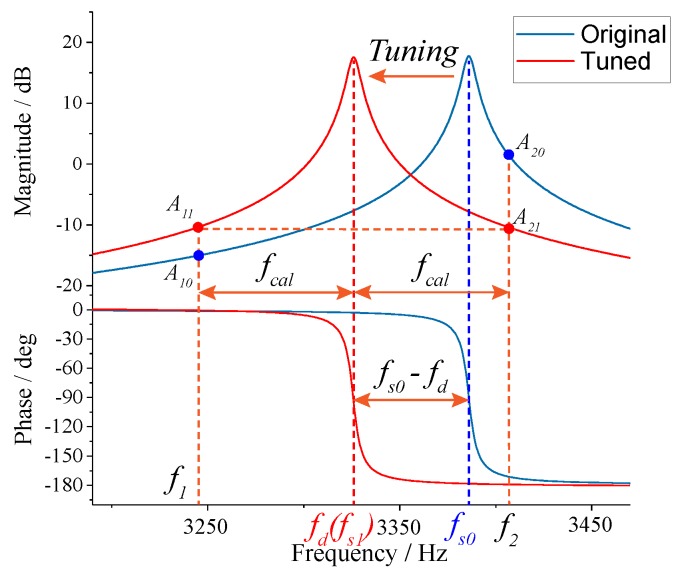
The schematic of the frequency tuning principle.

Figure 2 illustrates the schematic of the frequency tuning principle based on digital excitation-calibration technique, where *f${}_{d}$* is the drive-mode resonance frequency, and *f${}_{s0}$* and *f${}_{s1}$* are the natural and real-time resonance frequencies of sense mode, respectively. The frequency split between drive mode and the two-side excitation signal (*f${}_{1}$* and *f${}_{2}$*) is described as

(2) $$f_{cal}=f_{2}-f_{d}=f_{d}-f_{1}$$

The amplitude responses of *f${}_{1}$* and *f${}_{2}$* (*A${}_{10}$* and *A${}_{20}$*) have an obvious distinction before tuning. After the *f${}_{s1}$* is tuned to equal *f${}_{d}$*, i.e., mode-matching is accomplished, the difference between amplitude responses of *f${}_{1}$* and *f${}_{2}$* (*A${}_{11}$* and *A${}_{21}$*) will decrease to 0 due to the symmetry of the sense-mode dynamics. Therefore, the amplitude difference can reflect the degree of mode-matching. By integrating the amplitude difference, the DC tuning voltage is obtained.

## 3. Digital Excitation-Calibration Control System

### 3.1. System Design

**Figure 3:**
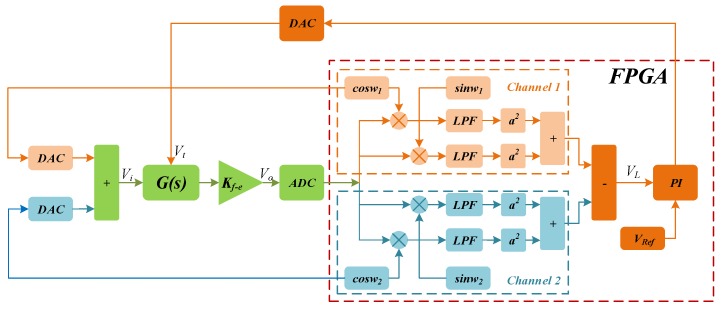
The block diagram of digital excitation-calibration system.

The block diagram of digital excitation-calibration control system for the closed-loop mode-matching is shown in Figure 3, where *V${}_{i}$* is the two-side excitation signal and *V${}_{i}$* = $\cos w_{1}t$ + $\cos w_{2}t$, *G*(*s*) represents the sense-mode dynamics of the MEMS gyroscope and *K${}_{f-e}$* is preamplifier gain. The output of preamplifier (*V${}_{o}$*) contains the amplitude response information of two-side excitation signal. The demodulation result (*V${}_{L}$*) of *V${}_{o}$* is fed into proportional integral (PI) controller to generate the DC tuning voltage (*V${}_{t}$*). Feedback of *V${}_{t}$* to sense dynamics contributes to building the closed control loop with the ability of automatic mode-matching.

The transfer function of sense mode applied with tuning voltage (*V${}_{t}$*) can be expressed as

(3) $$G\left(s\right)=\frac{\frac{1}{m_{s}}}{s^{2}+\frac{w_{s0}}{Q}s+w_{s0}^{2}-bV_{t}^{2}}$$

Based on Equation (3), the preamplifier output (*V${}_{o}$*) and demodulation result (*V${}_{L}$*) can be obtained as

(4) $$\left\{\begin{array}{c} V_{o}=a_{1}\cos\left(w_{1}t+\phi_{1}\right)+a_{2}\cos\left(w_{2}t+\phi_{2}\right)\\ V_{L}=\frac{1}{4}\left(a_{1}^{2}-a_{2}^{2}\right) \end{array}\right.$$

where *a${}_{1}$* and *a${}_{2}$* represent the amplitude responses of two channels excitation signals, while $\phi_{1}$ and $\phi_{2}$ are the phase responses, which can be further written as

(5) $$\left\{\begin{array}{l} a_{1}=\frac{\frac{K_{f-e}}{m_{s}}}{\sqrt{{\left(w_{s0}^{2}-bV_{t}^{2}-w_{1}^{2}\right)}^{2}+{\left(\frac{w_{1}w_{s0}}{Q}\right)}^{2}}}\\ \phi_{1}=arctg\left(\frac{w_{s0}^{2}-bV_{t}^{2}-w_{1}^{2}}{\frac{w_{1}w_{s0}}{Q}}\right)\\ a_{1}=\frac{\frac{K_{f-e}}{m_{s}}}{\sqrt{{\left(w_{s0}^{2}-bV_{t}^{2}-w_{2}^{2}\right)}^{2}+{\left(\frac{w_{2}w_{s0}}{Q}\right)}^{2}}}\\ \phi_{1}=arctg\left(\frac{w_{s0}^{2}-bV_{t}^{2}-w_{2}^{2}}{\frac{w_{2}w_{s0}}{Q}}\right) \end{array}\right.$$

In our previous work [30], another control loop called feedback calibration with an increased complexity was designed and analyzed. Such a scheme uses the two-side excitation signal and the feedback of preamplifier signal to actuate the sense mode at the same time. Consequently, it has a better immunity against the variation of sense-mode quality factor, which is further discussed in the following analysis. However, the feedback of preamplifier signal makes the stability of mode-matching control loop susceptible to the Coriolis acceleration. Therefore, the preamplifier signal feedback loop is cancelled in the proposed approach to achieve a better stability.

### 3.2. Open-Loop Analysis

#### 3.2.1. Frequency Limit of Excitation Signal

**Figure 4:**
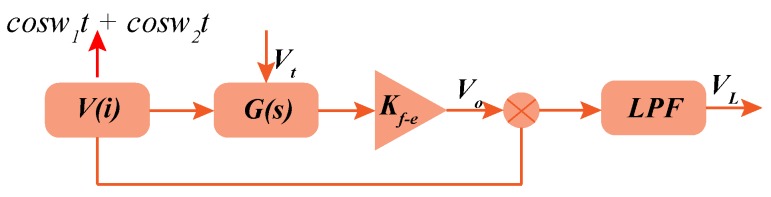
The simplified block diagram of open-loop system.

To study the characteristics of the closed-loop control system, open-loop analysis is deduced firstly. The simplified block diagram of open-loop system is shown in Figure 4.

The substituted open-loop tuning voltage of *V${}_{t}$* in the open-loop system is applied to sense mode to alter its resonant frequency. Through observing the demodulation result (*V${}_{L}$*), we can acquire the characteristics of the closed-loop system. Assuming the reference voltage of PI controller is set as 0 V, the stable DC tuning voltage in closed-loop system can be inferred from the Zero Crossing Point (ZCP) of the demodulation result (*V${}_{L}$*), because the PI controller will stop integrating once demodulation result (*V${}_{L}$*) reduces to 0 V.

According to Equations (4) and (5), the demodulation result can be further expressed as

(6) $$V_{L}=\frac{1}{4}\left[\frac{{\left(\frac{K_{f-e}}{m_{s}}\right)}^{2}}{{\left(w_{s0}^{2}-bV_{t}^{2}-w_{1}^{2}\right)}^{2}+{\left(\frac{w_{1}w_{s0}}{Q}\right)}^{2}}-\frac{{\left(\frac{K_{f-e}}{m_{s}}\right)}^{2}}{{\left(w_{s0}^{2}-bV_{t}^{2}-w_{2}^{2}\right)}^{2}+{\left(\frac{w_{2}w_{s0}}{Q}\right)}^{2}}\right]$$

**Figure 5:**
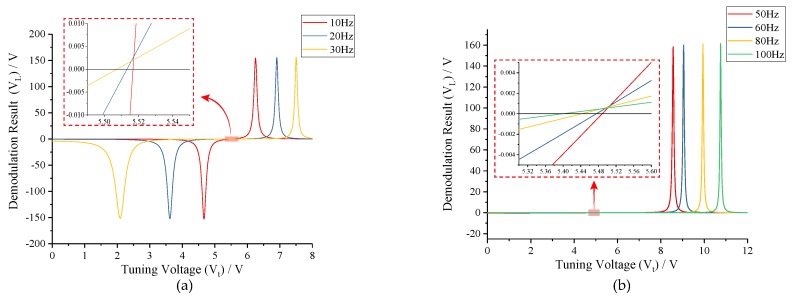
Demodulation results under different frequency splits: (**a**) *f${}_{cal}$* < *f${}_{s0}$* − *f${}_{d}$*; and (**b**) *f${}_{cal}$* > *f${}_{s0}$* − *f${}_{d}$*.

To determine the impacts of frequency split (*f${}_{cal}$*) between two-side excitation signal and drive mode on the tuning voltage, the relationship curves between *V${}_{L}$* and *V${}_{t}$* are drafted under conditions of *f${}_{cal}$* < *f${}_{s0}$* − *f${}_{d}$* and *f${}_{cal}$* > *f${}_{s0}$* − *f${}_{d}$*, respectively, which are shown in Figure 5. As can be seen in Figure 5a, each demodulation result (*V${}_{L}$*) experiences a resonance hump before arriving its ZCP under *f${}_{cal}$* < *f${}_{s0}$* − *f${}_{d}$* condition, which will lead to a saturation state of tuning voltage (*V${}_{t}$*) and the subsequent mode-mismatching in closed-loop system. The primary reason for this phenomenon is, in the process of tuning from *f${}_{s0}$* to *f${}_{d}$*, the sense-mode resonance frequency (*f${}_{s1}$*) will coincide with the right side of excitation signal (*f${}_{2}$*) sometime. When *f${}_{2}$* shares the same value with *f${}_{s1}$*, the amplitude response of *f${}_{2}$* will reach a peak value, thus maximizing the difference between the amplitude responses of *f${}_{1}$* and *f${}_{2}$*. Differently, each demodulation result (*V${}_{L}$*) monotonically increases to 0 with the raise of tuning voltage in Figure 5b. According to Equation (1) and the design parameters in Table 1, the perfect tuning voltage of the MEMS gyroscope can be calculated as 5.518 V. Apparently, four ZCPs under *f${}_{cal}$* > *f${}_{s0}$* − *f${}_{d}$* condition locate nearly to the perfect point. Therefore, the mode-matching condition can be achieved under *f${}_{cal}$* > *f${}_{s0}$* − *f${}_{d}$* condition with a minute deviation, i.e., *f${}_{cal}$*, *f${}_{s0}$* and *f${}_{d}$* in the closed-loop system should satisfy

(7) $$f_{cal}>f_{s0}-f_{d}$$

#### 3.2.2. Amplitude Ratio of Excitation Signal

In Figure 5b we show that, with the increase of frequency split (*f${}_{cal}$*), the ZCPs will be farther away from the perfect point in the case of *f${}_{cal}$* > *f${}_{s0}$* − *f${}_{d}$*. The tuning errors under different frequency splits are shown in Table 2.

The digital excitation-calibration technique takes advantage of the symmetry of the sense-mode dynamics. However, the sense-mode amplitude responses of symmetry points on both sides of the resonance peak are not actually absolutely symmetrical. To address the tuning errors stemming from this asymmetry, we use two channels signals with different amplitudes to actuate the sense mode. Setting the amplitudes as *K${}_{1}$* and *K${}_{2}$*, we can rewrite the demodulation result (*V${}_{L}$*) as

(8) $$V_{L}=\frac{1}{4}\left[{\left(K_{1}a_{1}\right)}^{2}-{\left(K_{2}a_{2}\right)}^{2}\right]$$

Assuming *V${}_{L}$* = 0 and considering *V${}_{t}$* = $\sqrt{\frac{w_{s0}^{2}-w_{d}^{2}}{b}}$, the amplitude ratio of two channels excitation signals can be derived as

(9) $$\frac{K_{1}}{K_{2}}=\frac{2w_{d}-w_{cal}}{2w_{d}+w_{cal}}=\frac{2f_{d}-f_{cal}}{2f_{d}+f_{cal}}$$

**Figure 6:**
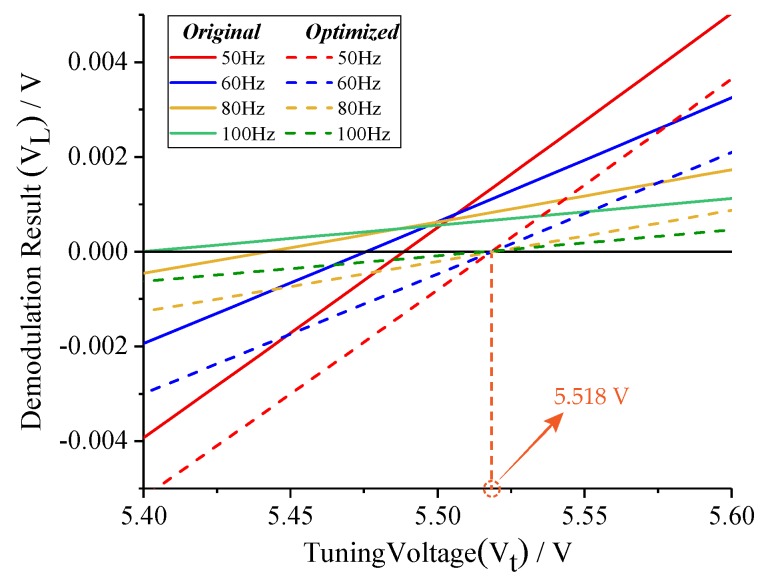
Comparison of demodulation results before and after optimizing.

According to Equation (9), we optimize the two-side excitation signal under the condition of *f${}_{cal}$* > *f${}_{s0}$* − *f${}_{d}$*. The comparison of demodulation results (*V${}_{L}$*) before and after optimizing is shown in Figure 6. Different from demodulation results (*V${}_{L}$*) without optimizing, new relationship curves perfectly converge at the theoretic point. Therefore, the tuning error can be effectively eliminated, and the effectiveness of amplitude optimizing is validated.

#### 3.2.3. Quality Factor of Sense Mode

**Figure 7:**
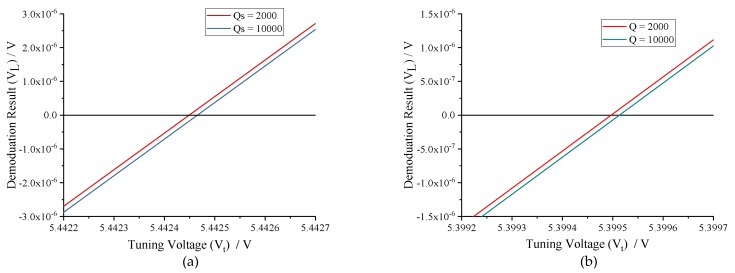
Comparison of demodulation results with different sense-mode Q-factors under conditions of *f${}_{cal}$* = 60 Hz and *f${}_{cal}$* =100 Hz: (**a**) *f${}_{cal}$* = 60 Hz; and (**b**) *f${}_{cal}$* =100 Hz.

The quality factor (Q-factor) of MEMS gyroscope has a direct impact on its mechanical sensitivity. To determine the influence of sense-mode Q-factor on tuning voltage, the relationship between *V${}_{L}$* and *V${}_{t}$* is analyzed under conditions of *Q${}_{s}$* = 2000 and *Q${}_{s}$* = 10,000. The frequency split (*f${}_{cal}$*) is set to 60 Hz and 100 Hz, respectively, and the analytical results are illustrated in Figure 7. Obviously, the tuning voltages with different sense-mode Q-factors are essentially equivalent, whether the *f${}_{cal}$* is near or far from the *f${}_{s0}$* − *f${}_{d}$*. It reveals that MEMS gyroscopes with *Q${}_{s}$* = 2000 and *Q${}_{s}$* = 10,000 nearly have the same tuning accuracy in case of the amplitude response of excitation signal can be precisely detected. However, in the practical circuit, the tuning control loop and the force feedback control loop share the same interface. To maintain the stabilization of the force feedback control loop when Q-factor is high, the preamplifier gain (*K${}_{f-e}$*) needs to be turned down. As a result, the output of preamplifier, which is used to demodulate the amplitude response of excitation signal, is hard to be detected. Moreover, the integral gain of PI controller has to be improved to shorten the tuning time. Therefore, an excessively high sense-mode Q-factor is not conducive to the digital excitation-calibration control.

### 3.3. Closed-Loop Analysis

Due to the nonlinearity of closed-loop control system, the average analysis method is exploited to analyze its stabilization [31]. According to Equation (3), the differential equation between the input of the system (*V${}_{i}$*) and the output of preamplifier (*V${}_{o}$*), demodulation result (*V${}_{L}$*) and output of PI controller (*V${}_{t}$*) can be, respectively, deduced as

(10) $$\left\{\begin{array}{l} \ddot{V_{o}}+\frac{w_{s0}}{Q}\dot{V_{o}}+\left(w_{s0}^{2}-bV_{t}^{2}\right)V_{o}=\frac{K_{f-e}}{m_{s}}V_{i}\\ \dot{V_{L}}=\beta \left(V_{o}V_{i}-V_{L}\right)\\ \dot{V_{t}}=K_{i}\left(V_{L}-C\right) \end{array}\right.$$

where β represents the cut-off frequency of low pass filter (LPF), and *K${}_{i}$* and *C* are the integral coefficient and reference voltage of PI controller, respectively. To analyze the transient properties of the closed-loop control system, we assume the input of system (*V${}_{i}$*) as $\cos\theta_{1}$ and $\theta_{1}$ = *w${}_{1}$t*. Then, the output of preamplifier (*V${}_{o}$*) and its derivative can be expressed as

(11) $$\left\{\begin{array}{c} V_{o}=a\cos\left(\theta_{1}+\phi \right)\\ \dot{V_{o}}=\dot{a}\cos\left(\theta_{1}+\phi \right)-aw_{1}\sin\left(\theta_{1}+\phi \right)-a\dot{\phi }\sin\left(\theta_{1}+\phi \right) \end{array}\right.$$

where *a* and ϕ, which can be considered as slowly-varying variables relative to angular velocity (*w*), are the transient amplitude and phase angle, respectively. Therefore, we suppose

(12) $$\dot{a}\cos\left(\theta_{1}+\phi \right)-a\dot{\phi }\sin\left(\theta_{1}+\phi \right)\equiv 0$$

Based on Equation (12) and *V${}_{i}$* = cos$\theta_{1}$, Equation (11) can be further deduced as

(13) $$\left\{\begin{array}{c} \dot{V_{o}}=-aw_{1}\sin\left(\theta_{1}+\phi \right)\\ \ddot{V_{o}}=\frac{K_{f-e}}{m_{s}}\cos\theta_{1}-\frac{w_{s0}}{Q}aw_{1}\sin\left(\theta_{1}+\phi \right)+\left(w_{s0}^{2}-bV_{t}^{2}\right)a\cos\left(\theta_{1}+\phi \right) \end{array}\right.$$

According to Equations (12) and (13), $\dot{a}$ and $\dot{\phi }$ can be extracted as

(14) $$\left\{\begin{array}{cc} \dot{a}= & \frac{1}{w_{1}}[\frac{K_{f-e}}{m_{s}}\cos\theta_{1}\sin\left(\theta_{1}+\phi \right)+aw_{1}^{2}\cos\left(\theta_{1}+\phi \right)\sin\left(\theta_{1}+\phi \right)\\ & +\frac{w_{s0}}{Q}aw_{1}\sin{\left(\theta_{1}+\phi \right)}^{2}-\left(w_{s0}^{2}-bV_{t}^{2}\right)a\cos\left(\theta_{1}+\phi \right)sin\left(\theta_{1}+\phi \right)]\\ \ddot{\phi }= & \frac{1}{aw_{1}}\left[aw_{1}^{2}\cos{\left(\theta_{1}+\phi \right)}^{2}+\frac{w_{s0}}{Q}aw_{1}\sin\left(\theta_{1}+\phi \right)\cos\left(\theta_{1}+\phi \right)\right]\\ & -\left(w_{s0}^{2}-bV_{t}^{2}\right)a\cos{\left(\theta_{1}+\phi \right)}^{2}+\frac{K_{f-e}}{m_{s}}\cos\theta_{1}\cos\left(\theta_{1}+\phi \right) \end{array}\right.$$

where *a* and ϕ are slowly-varying variables relative to $\theta_{1}$. Besides, cos(*$\theta_{1}$ + ϕ*) and sin(*$\theta_{1}$ + ϕ*) are the quasi-periodic signals. Therefore, $\dot{a}$ and $\dot{\phi }$ can be further evaluated with the average analysis method. Setting the integral interval as [−π, π], Equations (10) and (14) can be integrated and then rewritten as

(15) $$\left\{\begin{array}{l} \bar{\dot{a}}=-\frac{K_{f-e}}{2m_{s}w_{1}}\sin\bar{\phi }-\frac{w_{s0}}{2Q}\bar{a}\\ \bar{\dot{\phi }}=-\frac{K_{f-e}}{2m_{s}\bar{a}w_{1}}\cos\bar{\phi }+\frac{w_{s0}^{2}-b{\bar{V}}_{t}^{2}-w_{1}^{2}}{2w_{1}}\\ {\bar{\dot{V}}}_{L}=\beta \left(\frac{\bar{a}}{2}\cos\bar{\phi }-{\bar{V}}_{L}\right)\\ {\bar{\dot{V}}}_{t}=K_{i}\left({\bar{V}}_{L}-C\right) \end{array}\right.$$

Equation (15) is the average equation of the closed-loop excitation-calibration control system. It can be observed that the four variables ($\bar{a},\bar{\phi },{\bar{V}}_{L}\;\mathrm{and}\;{\bar{V}}_{t}$) are coupled to each other and the equation is still complicated and nonlinear. To simplify it further, location linearization is adopted around its equilibrium points. However, due to the intercoupling of four variables, the theoretic equilibrium points of the average equation are hard to solve directly. Therefore, we assume the unique equilibrium point as (${\bar{a}}_{0},{\bar{\phi }}_{0},{\bar{V}}_{L0}\;\mathrm{and}\;{\bar{V}}_{t0}$) and the linear Jacobian matrix acquired through analyzing the numerical equation is

(16) $$\frac{\partial f}{\partial {\bar{a},\bar{\phi },{\bar{V}}_{L},{\bar{V}}_{t}}_{\left({\bar{a}}_{0},{\bar{\phi }}_{0},{\bar{V}}_{L0},{\bar{V}}_{t0}\right)}}=\left[\begin{array}{cccc} -\frac{w_{s0}}{2Q} & -\frac{K_{f-e}}{2m_{s}w_{1}}\cos{\bar{\phi }}_{0} & 0 & 0\\ \frac{K_{f-e}}{2m_{s}w_{1}{\bar{a}}_{0}^{2}}\cos{\bar{\phi }}_{0} & \frac{K_{f-e}}{2m_{s}\bar{a}w_{1}}\sin{\bar{\phi }}_{0} & 0 & \frac{b{\bar{V}}_{t0}}{w_{1}}\\ \frac{\phi }{2}\cos{\bar{\phi }}_{0} & -\frac{\phi }{2}{\bar{a}}_{0}\sin{\bar{\phi }}_{0} & -\beta & 0\\ 0 & 0 & K_{i} & 0 \end{array}\right]$$

The characteristic equation of Equation (16) can be expressed as

(17) $$\lambda \left(\lambda -a_{33}\right)\cdot \left[\left(\lambda -a_{11}\right)\left(\lambda -a_{22}\right)-a_{12}a_{21}\right]+a_{43}a_{24}\cdot \left[\left(\lambda -a_{11}\right)a_{32}-a_{12}a_{31}\right]=0$$

where a${}_{xx}$ represent the elements of Jacobian matrix and the coefficients of $\lambda^{x}$ are

(18) $$\left\{\begin{array}{c} \lambda_{4}:c_{0}=1\\ \lambda_{3}:c_{1}=-a_{11}-a_{22}-a_{33}\\ \lambda_{2}:c_{2}=a_{11}\cdot a_{22}+a_{11}\cdot a_{33}+a_{22}\cdot a_{33}-a_{12}\cdot a_{21}\\ \lambda_{1}:c_{3}=-a_{11}\cdot a_{22}\cdot a_{33}+a_{12}\cdot a_{21}\cdot a_{33}-a_{43}\cdot a_{24}\cdot a_{32}\\ \lambda_{0}:c_{4}=a_{43}\cdot a_{24}\cdot a_{11}\cdot a_{32}-a_{11}\cdot a_{32}\cdot a_{12}\cdot a_{31} \end{array}\right.$$

According to Routh–Hurwitz stability criterion, a stable system should satisfy

(19) $$\left\{\begin{array}{c} \Delta_{1}=c_{1}\cdot c_{2}-c_{0}\cdot c_{3}\\ \Delta_{2}>c_{1}^{2}\cdot c_{4}/c_{3} \end{array}\right.$$

**Figure 8:**
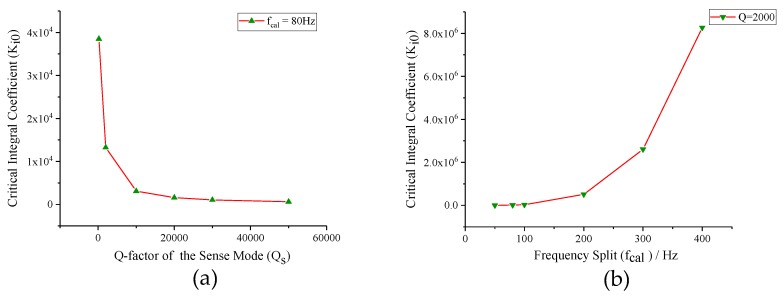
The relationship curves between the critical integral coefficient and key parameters of mode-matching system: (**a**) frequency split; and (**b**) Q-factor of the sense mode.

Equation (19) provides a theoretic guide to the design of the integral coefficient of PI controller (*K${}_{i}$*). Figure 8a illustrates the relationship between the critical integral coefficient (*K${}_{i0}$*) and the frequency split (*f${}_{cal}$*) based on the variable-control approach. Assuming sense-mode Q-factor as a constant, the critical integral coefficient (*K${}_{i0}$*) increases quickly with the expansion of frequency split (*f${}_{cal}$*). It indicates that the optional range of integral coefficient (*K${}_{i}$*) is wider when the frequency of excitation signal is farther away from sense-mode resonance frequency. Conversely, the critical integral coefficient (*K${}_{i0}$*) is negatively correlated to the sense-mode Q-factor (*Q${}_{s}$*) when the frequency split (*f${}_{cal}$*) remains unchanged, which is shown in Figure 8b. It reveals that the integral coefficient (*K${}_{i}$*) needs to be turned down to maintain the stabilization of the mode-matching control loop when Q-factor is high.

## 4. Simulation

**Figure 9:**
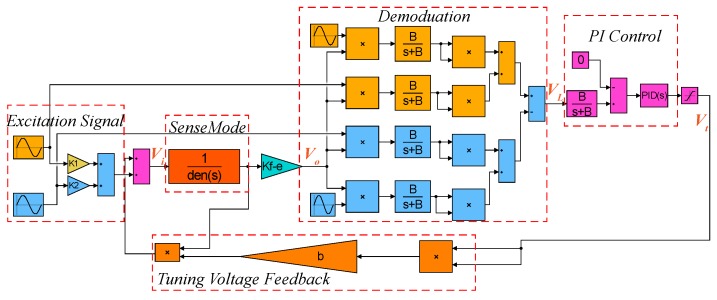
System model of the closed-loop excitation-calibration control system constructed by SIMULINK.

To verify the feasibility of closed-loop excitation-calibration control system and the validity of theoretic analysis, the system simulation was implemented by SIMULINK. The system model constructed in SIMULINK is shown in Figure 9.

**Figure 10:**
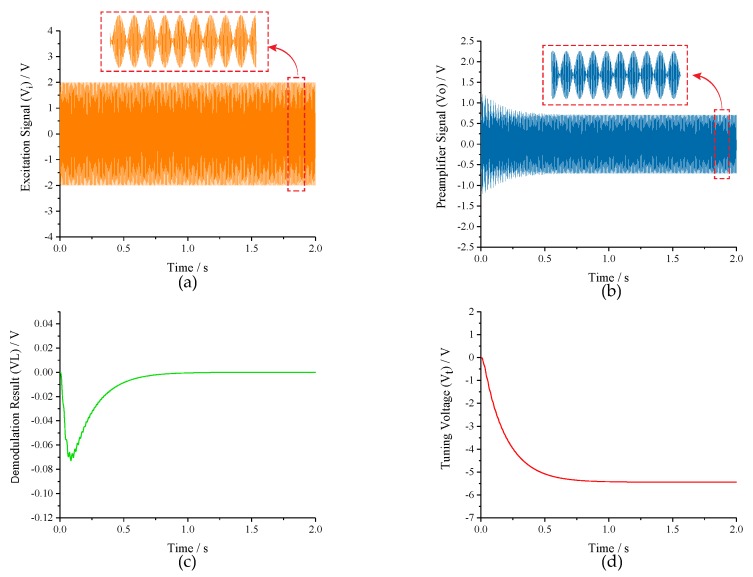
The transient responses of the closed-loop excitation-calibration control system: (**a**) excitation signal; (**b**) preamplifier signal; (**c**) demodulation result; and (**d**) tuning voltage.

The simulation parameters of the MEMS gyroscope were consistent with the parameters in Table 1. The preamplifier gain (*K${}_{f-e}$*), cut-off frequency of low-pass filter (LPF) and frequency split (*f${}_{cal}$*) were set to 10, 50 Hz and 80 Hz, respectively. Besides, the *K${}_{p}$* and *K${}_{i}$* of PI controller were assigned to 20 and 3000 to meet the stability constraints of Equation (19). The transient responses of the closed-loop excitation-calibration control system are shown in Figure 10. Figure 10a is the waveform of excitation signal, which is the superposition of two channels signals with different frequencies. The output of preamplifier (*V${}_{o}$*) in Figure 10b contains the amplitude response information of excitation signal, and the amplitude difference between two excitation signals is extracted by subsequent square demodulation. As the demodulation result (*V${}_{L}$*) in Figure 10c stabilizes to 0 within 1 s, the tuning voltage (*V${}_{t}$*) in Figure 10d gradually levels off to a constant which coincides with the analytical result in Table 2, indicating that the micro-gyroscope is in mode-matched operation.

**Figure 11:**
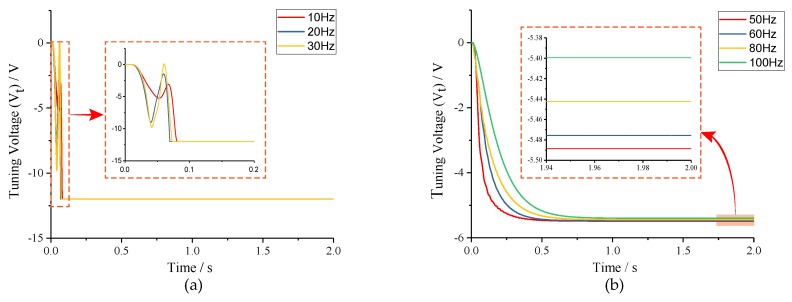
Tuning voltages under different conditions: (**a**) *f${}_{cal}$* < *f${}_{s0}$* − *f${}_{d}$*; and (**b**) *f${}_{cal}$* > *f${}_{s0}$* − *f${}_{d}$*.

To verify that tuning accuracy is relevant to the frequency split between the drive mode and two-side excitation signal (*f${}_{cal}$*), different values of *f${}_{cal}$* were selected under conditions of *f${}_{cal}$* < *f${}_{s0}$* − *f${}_{d}$* and *f${}_{cal}$* > *f${}_{s0}$* − *f${}_{d}$*, respectively. As can be seen from the simulation results in Figure 11a, when *f${}_{cal}$* < *f${}_{s0}$* − *f${}_{d}$*, the tuning voltages reach the saturation condition quickly, and mode-matching is not realized. In Figure 11b, when *f${}_{cal}$* > *f${}_{s0}$* − *f${}_{d}$*, the tuning voltages stabilize nearly to the theoretic value within 1s. From the partially enlarged view, the simulation tuning voltages can be read out specifically and the tuning errors can be obtained subsequently, which are shown in Table 3. Compared with analytical results in Table 2, nearly no difference presents, which certifies the correctness of open-loop analysis.

**Figure 12:**
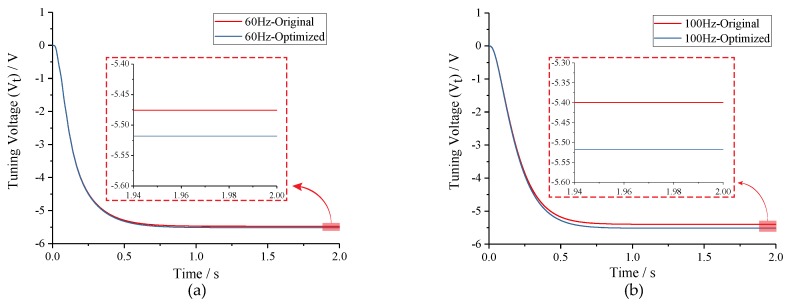
Comparison of tuning voltages with different amplitude ratios of the excitation signal under conditions of *f${}_{cal}$* = 60 Hz and *f${}_{cal}$* =100 Hz: (**a**) *f${}_{cal}$* = 60 Hz; and (**b**) *f${}_{cal}$* =100 Hz.

To test the optimization effect of Equation (9), the amplitude ratio of two-side excitation signal was set to 1:1 and (2*f${}_{d}$* − *f${}_{cal}$*):(2*f${}_{d}$* + *f${}_{cal}$*), respectively, under conditions of *f${}_{cal}$* = 60 Hz and *f${}_{cal}$* = 100 Hz. Simulation results in Figure 12 indicate that the tuning voltages can be adjusted to the theoretic value by amplitude optimizing regardless of the tuning accuracy before optimizing.

**Figure 13:**
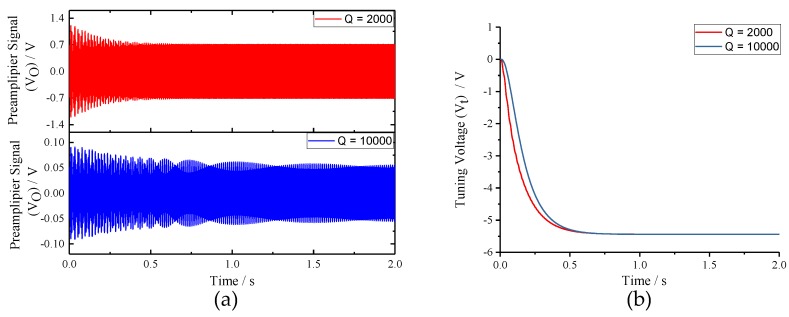
The key signal waveforms of the excitation-calibration control system with different Q-factors: (**a**) output signal of preamplifier; and (**b**) tuning voltage.

To verify the impacts of sense-mode Q-factor (*Q${}_{s}$*) on the mode-matching system, *Q${}_{s}$* was assigned to 2000 and 10,000, respectively, under the condition of *f${}_{cal}$* = 60 Hz. Waveforms of key signals in the closed-loop excitation-calibration control system are presented in Figure 13. Figure 13b reveals that the final tuning voltages under *Q${}_{s}$* = 2000 and *Q${}_{s}$* = 10,000 conditions are basically equivalent, which coincides with the open-loop analysis. However, when *Q${}_{s}$* = 10,000, the output of preamplifier (*V${}_{o}$*) is excessively weak, which makes it rather hard to detect with the available performance of practical circuit. Meanwhile, to shorten the tuning time, the integral coefficient (*K${}_{i}$*) needs to be improved, which will lead to the enhancement of system instability according to the closed-loop analysis above. Therefore, it can be concluded that the work interval of the integral coefficient (*K${}_{i}$*) will be very narrow when sense-mode Q-factor (*Q${}_{s}$*) is high.

**Figure 14:**
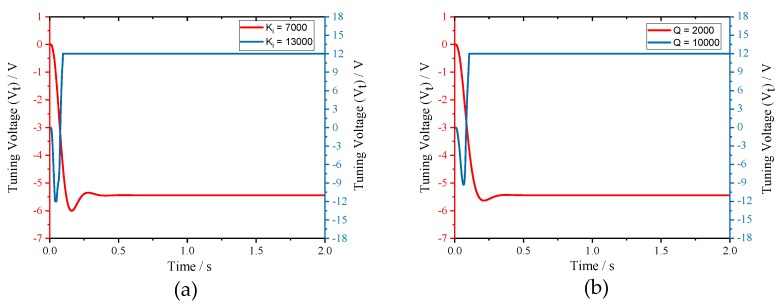
Stability tests of the excitation-calibration control system: (**a**) tuning voltages with the same *Q${}_{s}$*, *f${}_{cal}$* and different *K${}_{i}$*; and (**b**) tuning voltages with the same *K${}_{i}$*, *f${}_{cal}$* and different *Q${}_{s}$*.

Finally, the stability analysis was verified. We set integral coefficient (*K${}_{i}$*) to 7000 and 13,000 under *Q${}_{s}$* = 2000, *f${}_{cal}$* = 80 Hz (*K${}_{i0}$* = 10,606) condition firstly. Then, the integral coefficient (*K${}_{i}$*) was set to 4000 under *Q${}_{s}$* = 2000, *f${}_{cal}$* = 80 Hz (*K${}_{i0}$* = 10,606) and *Q${}_{s}$* = 10,000, *f${}_{cal}$* = 80 Hz (*K${}_{i0}$* = 2487) conditions, respectively. The response processes of tuning voltage are shown in Figure 14. From the simulation results, we can find that, when *Q${}_{s}$*, *f${}_{cal}$* and *K${}_{i}$* satisfy the constraint of Equation (19), the closed control loop can fulfill mode-matching effectively. Otherwise, the tuning voltage will get saturated quickly and mode-matching cannot be realized.

## 5. Experiment

**Figure 15:**
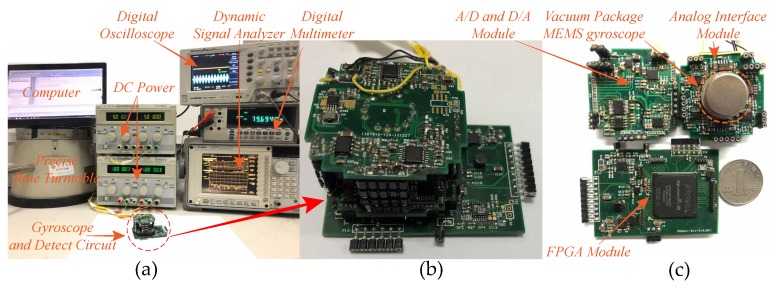
The experimental platform: (**a**) the experimental facilities; (**b**) the assembled control circuit; and (**c**) the three sub-modules of the control circuit.

A series of experiments was carried out to verify the theoretic analysis and simulation. Figure 15 shows the experimental platform in laboratory environment. The involved experimental facilities contain two DC powers, a digital multimeter, a digital oscilloscope, a computer, a precise rate tunable and a dynamic signal analyzer, as shown in Figure 15a. The assembled control circuits used for system control and signals detection are presented in Figure 15b. Three sub-modules of the control circuits are the analog interface module, the A/D and D/A module and the FPGA module, which are illustrated in Figure 15c. The entire control system consists of four closed loops: self-excitation drive loop, force feedback control loop, quadrature error correction loop and the excitation-calibration control loop. All the control algorithms are implemented in the FPGA chip. To ensure enough logical source, we adopted the EP3C55F484I7 chip from the Altera corporation, which contains 55,850 programmable logical units, 239,600 RAM bits and 156 multipliers. The DAC is CS4344 chip from CURRUS corporation and ADC is AD7767 chip from ADI corporation. Both sampling rates were set to 48 kHz to ensure enough working rate of the entire control system.

**Figure 16:**
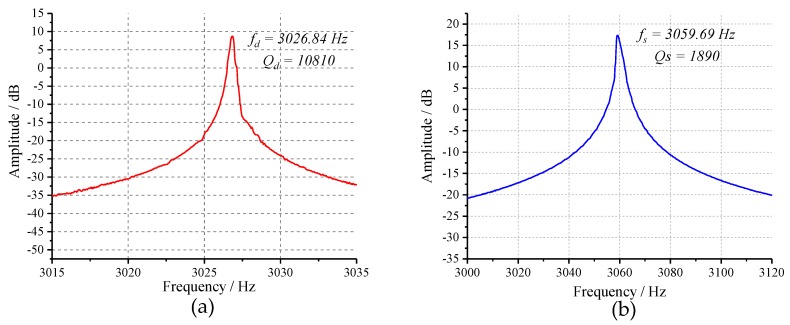
Results of the open-loop sweep-frequency experiment: (**a**) the drive mode; and (**b**) the sense mode.

**Figure 17:**
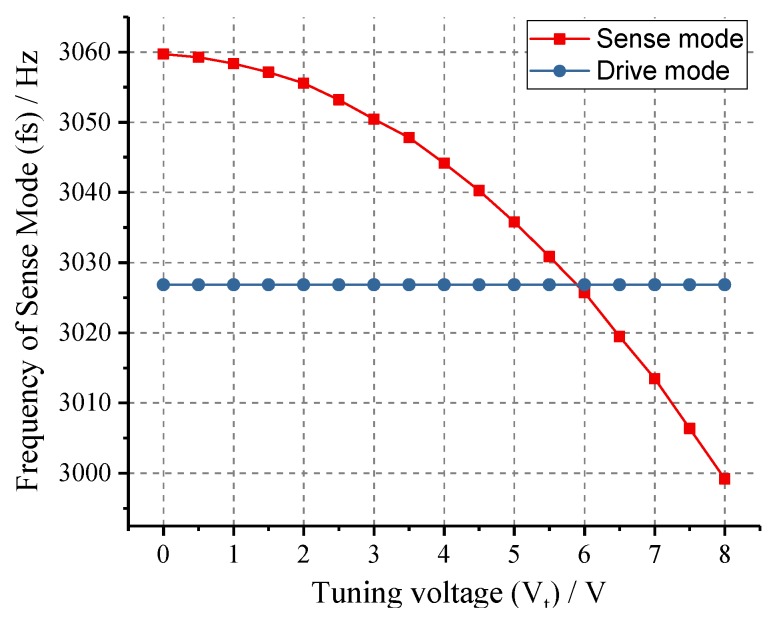
The experimental relationship curves between the resonance frequencies of two operating modes and tuning voltage.

The open-loop frequency response experiment was performed firstly to determine the resonance frequencies of drive and sense modes of the tested prototype. Experimental results in Figure 16 indicate that the device has a drive-mode frequency of 3026.84 Hz with a Q-factor of 10,810 and a sense-mode frequency of 3059.69 Hz with a Q-factor of 1890. To verify the tuning capability of tuning mechanism, 0–8 V DC voltages were applied to the tuning electrodes with a step of 0.5 V. The relationship between the resonance frequencies of two operating modes and tuning voltage are illustrated in Figure 17. The experimental curves demonstrate that the sense-mode resonance frequency decreases monotonously with the increase of tuning voltage while drive mode keeps unchanged, which is consistent with the theoretic analysis of Equation (1). Through fitting the experimental data by MATLAB, the mechanical parameter (*b*) and perfect tuning voltage (*V${}_{t}$*) of this tested prototype can be obtained as 227,540 and −5.889 V.

**Figure 18:**
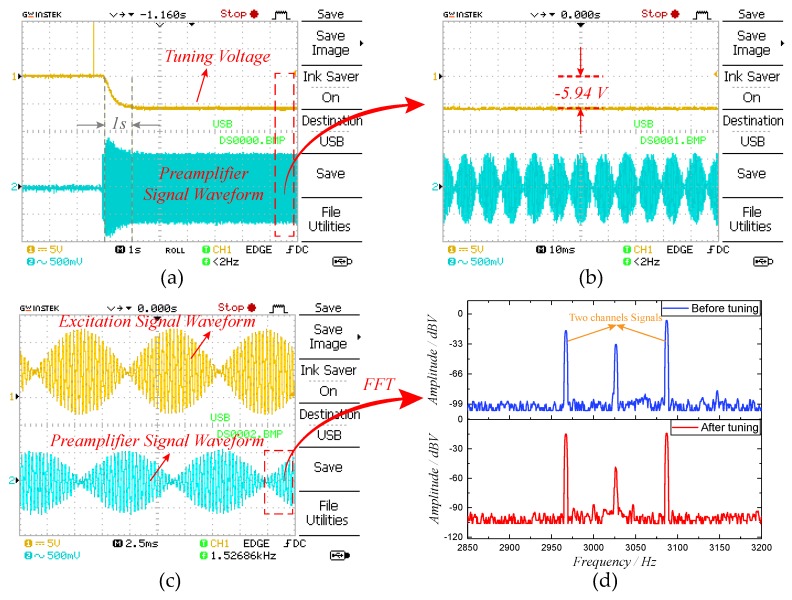
The key signal waveforms of the excitation-calibration control system: (**a**) the transient responses of tuning voltage and preamplifier signal; (**b**) the enlarged view of tuning voltage and preamplifier signal in steady state; (**c**) the enlarged view of excitation signal and preamplifier signal in steady state; and (**d**) the spectrums of preamplifier signal under conditions of mode-matching and mode-mismatching.

To verify the tuning effect of the excitation-calibration control system, the closed-loop control experiment was carried out. The frequency split (*f${}_{cal}$*) of excitation signal was set to 60 Hz and the amplitude ratio was optimized according to Equation (9). The key signal waveforms of the mode-matching system are measured in Figure 18. It can be observed in Figure 18a that the tuning voltage and the preamplifier signal reach the steady state within approximate 1 s, indicating that the mode-matching is realized. Through the partially enlarged view in Figure 18b, we find that the final tuning voltage is −5.94 V and the corresponding resonant frequency of sense mode is 3026.28 Hz. Thus, the excitation-calibration control system can achieve mode-matching condition with an error of 0.56 Hz. Figure 18c is the enlarged view of the two-side excitation signal and preamplifier signal, both of which are consistent with the simulation results in Figure 10. The spectrums of preamplifier signal are illustrated in Figure 18d. The amplitude responses of two channels signals have an obvious distinction before tuning, however, they become essentially equivalent when the tested prototype is in mode-matched operation. It coincides with the tuning principle in Figure 2 and proves the feasibility of digital excitation-calibration technique. Simultaneously, the spectrums reveal that the output of sense-mode dynamics mainly contains three channels signals, which are two channels excitation signals and the Coriolis signal.

**Figure 19:**
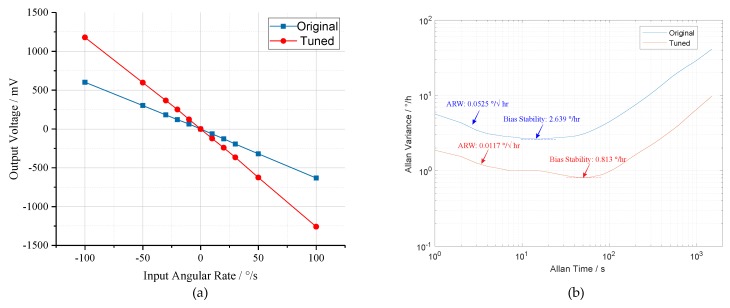
Results of performance experiments under conditions of mode-matching and mode-mismatching: (**a**) the output–input relationship curves; and (**b**) the Allen variance curves.

Finally, the performance of the prototype was tested. The output–input relationship curves are shown in Figure 19a. Obviously, in the conditions of mode-matching and mode-mismatching, the tested prototype exhibits a scale factor of −6.19 mV/${}^{\circ }$/s and −12.21 mV/${}^{\circ }$/s, respectively, under the input angular rate range of ±100${}^{\circ }$/s. Subsequently, the drift characteristics of prototype were evaluated at room temperature. Comparison of the Allen Variance curves under mode-matched and mode-mismatched states is illustrated in Figure 19b. Compared to the mode-mismatched state, the bias instability of mode-matched prototype decreases from 2.639${}^{\circ }$/h to 0.813${}^{\circ }$/h and the Angular Random Walk (ARW) reduces from 0.0525${}^{\circ }$/$\sqrt{h}$ to 0.0117${}^{\circ }$/$\sqrt{h}$. Therefore, it can be concluded that the bias instability and the ARW of the prototype are improved by 3.25 and 4.49 times by mode-matching control. The above experimental results substantiate the effectiveness of mode-matching technology in terms of enhancing the mechanical sensitivity and bias stability of MEMS gyroscope.

## 6. Conclusions

This paper provides a digital excitation-calibration technique of dual-mass MEMS gyroscope for closed-loop mode-matching control. The gyroscope structure with the tuning mechanism and the principle of digital excitation-calibration technique are introduced firstly. Then, the design of digital excitation-calibration control system is introduced followed by open-loop and closed-loop analysis. The open-loop analysis focuses on the sources of tuning error. Analytical results reveal that frequency split between the two-side excitation signal and drive signal has a significant influence on the tuning accuracy due to the asymmetry of the sense-mode dynamics. To eliminate the tuning error, an optimized amplitude ratio of the two-side excitation signal is utilized to offset the asymmetry. The closed-loop analysis emphasizes on the stability of digital excitation-calibration control system. It indicates that the system stability and sense-mode Q-factor appear in a negative correlation. A system simulation was deduced by SIMULINK to verify the effectiveness of digital excitation-calibration system and theoretical analysis. The simulation results are proved to be consistent with the theoretical analysis. Experiments were carried out to evaluate the performance of prototype. The experimental results demonstrate that the digital excitation-calibration control system can realize mode-matching in 1 s with a tuning error of 0.56 Hz. Compared to mode-mismatched state, the bias instability and the ARW of prototype in mode-matched operation are enhanced by 3.25 times and 4.49 times, respectively.

## Figures and Tables

**Table 1:** The design parameters of dual-mass MEMS gyroscope.

Parameter	Value	Unit
Drive mode resonant frequency (*w${}_{s0}$*)	3345 × 2π	rad/s
Drive mode quality factor (*Q${}_{d}$*)	10,000	
Sense mode resonant frequency (*w${}_{d}$*)	3380 × 2π	rad/s
Sense mode quality factor (*Q${}_{s}$*)	2000	
Sense effective mass (*m${}_{s}$*)	9.02 × 10${}^{-7}$	Kg
Stiffness of sense structure (*k${}_{y}$*)	242.30	N/m
Structure thickness (*h*)	80	μm
Number of tuning combs (*n*)	92	
Tuning comb length (*l*)	180	μm
Tuning comb gap (*e*)	4.4	μm
Vacuum permittivity (ε)	8.854 × 10${}^{-12}$	F/m
Tuning comb mechanical parameter (*b*)	3.0518 × 10${}^{5}$	N/(mV${}^{2}$)

**Table 2:** Tuning errors under different frequency splits in open-loop analysis.

Frequency Split (Hz)	Analytic Tuning Voltage (V)	Tuning Error (Hz)
50	5.489	0.369
60	5.476	0.534
80	5.442	0.962
100	5.399	1.500

**Table 3:** Tuning errors under different frequency splits in open-loop analysis.

Frequency Split (f${}_{cal}$) (Hz)	Tuning Voltage (V)	Tuning Error (Hz)
50	−5.489	0.369
60	−5.476	0.534
80	−5.442	0.975
100	−5.399	1.488

## References

[1] Xia D., Yu C., Kong L. (2014). The development of micromachined gyroscope structure and circuitry technology. Sensors.

[2] Liu K., Zhang W., Chen W., Li K., Dai F., Cui F., Wu X., Ma G., Xiao Q. (2009). The development of micro-gyroscope technology. J. Micromech. Microeng..

[3] Passaro V., Cuccovillo A., Vaiani L., De Carlo M., Campanella C.E. (2017). Gyroscope technology and applications: A review in the industrial perspective. Sensors.

[4] Ho C.Y., Lee F.Y., Fang W. Development of a CMOS-MEMS gyroscope using pure-oxide and symmetric metal-oxide stacking structures. Proceedings of the 19th International Conference on Solid-State Sensors, Actuators and Microsystems (TRANSDUCERS).

[5] Cao H., Zhang Y., Han Z., Shao X., Gao J., Huang K., Shi Y., Tang J., Shen C., Liu J. (2019). Pole-Zero Temperature Compensation Circuit Design and Experiment for Dual-Mass MEMS Gyroscope Bandwidth Expansion. IEEE/ASME Trans. Mechatron..

[6] Zhanshe G., Fucheng C., Boyu L., Le C., Chao L., Ke S. (2015). Research development of silicon MEMS gyroscopes: A review. Microsyst. Technol..

[7] Prikhodko I.P., Nadig S., Gregory J.A., Clark W.A., Judy M.W. Half-a-month stable 0.2 degree-per-hour mode-matched MEMS gyroscope. Proceedings of the IEEE International Symposium on Inertial Sensors and Systems (INERTIAL).

[8] Rahafrooz A., Serrano D.E., Jafri I. (2018). Method and Apparatus for Electrostatic Mode-Alignment on Planar MEMS Gyroscopes. U.S. Patent.

[9] Sonmezoglu S., Alper S.E., Akin T. (2014). An automatically mode-matched MEMS gyroscope with wide and tunable bandwidth. J. Microelectromech. Syst..

[10] Xiao D., Zhou X., Li Q., Hou Z., Xi X., Wu Y., Wu X. (2016). Design of a disk resonator gyroscope with high mechanical sensitivity by optimizing the ring thickness distribution. J. Microelectromech. Syst..

[11] Sharma A., Zaman M.F., Ayazi F. (2009). A Sub-0.2^∘^/hr Bias Drift Micromechanical Silicon Gyroscope With Automatic CMOS Mode-Matching. IEEE J. Solid-State Circuits.

[12] Gando R., Kubo H., Masunishi K., Tomizawa Y., Ogawa E., Maeda S., Hatakeyama Y., Itakura T., Ikehashi T. A catch-and-release drive MEMS gyroscope with enhanced sensitivity by mode-matching. Proceedings of the IEEE International Symposium on Inertial Sensors and Systems (INERTIAL).

[13] Yang C., Li H. (2015). Digital control system for the MEMS tuning fork gyroscope based on synchronous integral demodulator. IEEE Sens. J..

[14] Joachim D., Lin L. (2003). Characterization of selective polysilicon deposition for MEMS resonator tuning. J. Microelectromech. Syst..

[15] Remtema T., Lin L. (2001). Active frequency tuning for micro resonators by localized thermal stressing effects. Sens. Actuators Phys..

[16] Wang K., Wong A.C., Hsu W.T., Nguyen C.C. Frequency trimming and Q-factor enhancement of micromechanical resonators via localized filament annealing. Proceedings of the International Solid State Sensors and Actuators Conference (Transducers’ 97).

[17] Jia J., Ding X., Gao Y., Li H. (2018). Automatic Frequency Tuning Technology for Dual-Mass MEMS Gyroscope Based on a Quadrature Modulation Signal. Micromachines.

[18] Gallacher B.J., Hedley J., Burdess J.S., Harris A.J., Rickard A., King D.O. (2005). Electrostatic correction of structural imperfections present in a microring gyroscope. J. Microelectromech. Syst..

[19] Yesil F., Alper S., Akin T. An automatic mode matching system for a high Q-factor MEMS gyroscope using a decoupled perturbation signal. Proceedings of the Transducers-2015 18th International Conference on Solid-State Sensors, Actuators and Microsystems (TRANSDUCERS).

[20] Kim D.J., M’Closkey R.T. (2006). A systematic method for tuning the dynamics of electrostatically actuated vibratory gyros. IEEE Trans. Control. Syst. Technol..

[21] Keymeulen D., Fink W., Ferguson M.I., Peay C., Oks B., Terrile R., Yee K. (2005). Evolutionary Computation Applied to the Tuning of MEMS Gyroscopes.

[22] Keymeulen D., Ferguson M.I., Breuer L., Peay C., Oks B., Kim D., MacDonald E., Foor D., Terrile R., Yee K. Tuning of MEMS gyroscope using evolutionary algorithm and “switched drive-angle” method. Proceedings of the IEEE Aerospace Conference.

[23] Sung S., Sung W.T., Kim C., Yun S., Lee Y.J. (2009). On the mode-matched control of MEMS vibratory gyroscope via phase-domain analysis and design. IEEE/ASME Trans. Mechatron..

[24] Chang B.S., Sung W.T., Lee J.G., Lee K.Y., Sung S. Automatic mode matching control loop design and its application to the mode matched MEMS gyroscope. Proceedings of the IEEE International Conference on Vehicular Electronics and Safety.

[25] He C., Zhao Q., Liu D., Dong L., Yang Z., Yan G. An automatic real-time mode-matching MEMS gyroscope with fuzzy and neural network control. Proceedings of the Transducers & Eurosensors XXVII: The 17th International Conference on Solid-State Sensors, Actuators and Microsystems (TRANSDUCERS & EUROSENSORS XXVII).

[26] He C., Zhao Q., Huang Q., Liu D., Yang Z., Zhang D., Yan G. (2015). A MEMS vibratory gyroscope with real-time mode-matching and robust control for the sense mode. IEEE Sens. J..

[27] Ezekwe C.D., Boser B.E. (2008). A Mode-Matching ΣΔ Closed-Loop Vibratory Gyroscope Readout Interface with a 0.004^∘^/s/√Hz Noise Floor Over a 50 Hz Band. IEEE J. Solid-State Circuits.

[28] Marx M., Cuignet X., Nessler S., De Dorigo D., Manoli Y. (2019). An Automatic MEMS Gyroscope Mode Matching Circuit Based on Noise Observation. IEEE Trans. Circuits Syst. II Exp. Briefs.

[29] Yang B., Wang X., Deng Y., Hu D. (2016). Mechanical coupling error suppression technology for an improved decoupled dual-mass micro-gyroscope. Sensors.

[30] Wu L., Yang B., Wang G. Design of an Automatic Mode-matching System for MEMS Gyroscope. Proceedings of the Seventh International Conference on Instrumentation & Measurement, Computer, Communication and Control, IMCCC.

[31] Pedicini C., Iannelli L., Vasca F. The averaging method for control design and stability analysis of practical switched systems. Proceedings of the IEEE International Conference on Control Applications.

